# Supplementary material for: Mapping the sensing spots of aerolysin for single oligonucleotides analysis
Source: Nat Commun. 2018 Jul 19;9:2823. doi: 10.1038/s41467-018-05108-5 (PMC6053387; doi:10.1038/s41467-018-05108-5)
Supplement: Supplementary file 1 — Supplementary Information [file 41467_2018_5108_MOESM1_ESM.pdf]

Mapping the sensing spots of aerolysin nanopore for single oligonucleotides analysis

Chan Cao, Meng-Yin Li, Nuria Cirauqui, Ya-Qian Wang, Matteo Dal Peraro*, He Tian, Yi-Tao Long*

Supplementary Methods

All oligonucleotides used in this article were synthesized and purified by Sangon Biotech Co., Ltd. (Shanghai, China). Trypsin-EDTA solution, potassium chloride (≥99%) and decane (anhydrous, ≥99%) were purchased from Sigma-Aldrich Co., Ltd. (St. Louis, MO, USA). 1,2-diphytanoyl-*sn*-glycero-3-phosphocholine (powder, ≥99%) was obtained from Avanti Polar Lipids, Inc. (Alabaster, AL, USA). Ethylenediaminetetraacetic acid (EDTA, ≥99%) and Tris(hydroxymethyl)aminomethane (TRIS, ≥99%) were ordered from Aladdin Chemistry Co., Ltd. (Shanghai, China). Proaerolysin was received from Aerohead Scientific, Ltd. (Saskatoon, SK, Canada). All solutions were prepared by ultrapure water (18.2 MΩ·cm at 25 °C) using a simplicity water purification system from Merck Millipore (Bayswater, VIC, Australia).

Supplementary Tables

Supplementary Table 1. Main interactions observed between dA_{14} and the aerolysin pore lumen residues.

DNA base*	Pore lumen residues
14	R282, D216
13	D216, S280, T218
12	T218, S278, R220
11	R220
10	D222, S272
9	S272, N226
8	N226, Q268
7	T232, S261
6	T232, S261
5	N262
4	N262, K238
3	K238, E258
2	E258
1	K242, k244, E254

*DNA bases are numbered from 1 (3’) to 14 (5’).

Supplementary Table 2. Sequences of the oligonucleotides used in this article.

Oligonucleotide	Sequence (3' to 5')*
**dA$_{10}$**	AAAAAAAAAA
**dA$_{11}$**	AAAAAAAAAAA
**dA$_{12}$**	AAAAAAAAAAAA
**dA$_{13}$**	AAAAAAAAAAAAA
**dA$_{14}$**	AAAAAAAAAAAAAA
**dA$_{15}$**	AAAAAAAAAAAAAAA
**dA$_{16}$**	AAAAAAAAAAAAAAAA
**dA$_{17}$**	AAAAAAAAAAAAAAAAA
**dA$_{18}$**	AAAAAAAAAAAAAAAAAA
**dA$_{19}$**	AAAAAAAAAAAAAAAAAAA
**dA$_{20}$**	AAAAAAAAAAAAAAAAAAAA
**dA$_{14}$X1**	XAAAAAAAAAAAAA
**dA$_{14}$X2**	AXAAAAAAAAAAAA
**dA$_{14}$X3**	AAXAAAAAAAAAAA
**dA$_{14}$X4**	AAAXAAAAAAAAAA
**dA$_{14}$X5**	AAAAXAAAAAAAAA
**dA$_{14}$X6**	AAAAAXAAAAAAAA
**dA$_{14}$X7**	AAAAAAXAAAAAAA
**dA$_{14}$X8**	AAAAAAAXAAAAAA
**dA$_{14}$X9**	AAAAAAAAXAAAAA
**dA$_{14}$X10**	AAAAAAAAAXAAAA
**dA$_{14}$X11**	AAAAAAAAAAXAAA
**dA$_{14}$X12**	AAAAAAAAAAAXAA
**dA$_{14}$X13**	AAAAAAAAAAAAXA
**dA$_{14}$X14**	AAAAAAAAAAAAAX
**dA$_{14}$X4-A**	AAAAAAAAAAAAAA
**dA$_{14}$X4-C**	AAACAAAAAAAAAA
**dA$_{14}$X4-G**	AAAGAAAAAAAAAA
**dA$_{14}$X4-T**	AAATAAAAAAAAAA
**dA$_{14}$X11-A**	AAAAAAAAAAAAAA
**dA$_{14}$X11-C**	AAAAAAAAAACAAA
**dA$_{14}$X11-G**	AAAAAAAAAAGAAA
**dA$_{14}$X11-T**	AAAAAAAAAATAAA
**dA$_{14}$X11-^{m}C**	AAAAAAAAAA^{m}CAAA
**dA$_{14}$X11-^{o}G**	AAAAAAAAAA^{o}GAAA
**SP$_{14}$X11-A**	AGGCAATCAAAGGA
**SP$_{14}$X11-C**	AGGCAATCAACGGA
**SP$_{14}$X11-G**	AGGCAATCAAGGGA
**SP$_{14}$X11-T**	AGGCAATCAATGGA

* X in the sequence represents base deletion.

Supplementary Table 3. Detailed description of the equilibration protocol used for MD Simulations.

Equilibration step	Ensemble	Time step	Simulation time	Force Constant For Harmonic Restraints[a]
1	NVT	0.001 ps	25 ps	*BB*[b]: *10.0*<br>*SC*[c]: *5.0*<br>*L*[d]: *2.5*
2	NVT	0.001 ps	25 ps	*BB*[b]: *10.0*<br>*SC*[c]: *5.0*<br>*L*[d]: *2.5*
3	NPT	0.001 ps	25 ps	*BB*[b]: *10.0*<br>*SC*[c]: *5.0*<br>*L*[d]: *1.0*
4	NPT	0.002 ps	100 ps	*BB*[b]: *10.0*<br>*SC*[c]: *5.0*<br>*L*[d]: *0.5*
5	NPT	0.002 ps	100 ps	*BB*[b]: *10.0*<br>*SC*[c]: *5.0*<br>*L*[d]: *0.1*
6	NPT	0.002 ps	100 ps	*BB*[b]: *10.0*<br>*SC*[c]: *5.0*<br>*L*[d]: *0*
7	NPT	0.002 ps	100 ps	*BB*[b]: *10.0*<br>*SC*[c]: *5.0*<br>*L*[d]: *0*
8	NPT	0.002 ps	100 ps	*BB*[b]: *5.0*<br>*SC*[c]: *2.5*<br>*L*[d]: *0*
9	NPT	0.002 ps	100 ps	*BB*[b]: *2.5*<br>*SC*[c]: *1.0*<br>*L*[d]: *0*
10	NPT	0.002 ps	100 ps	*BB*[b]: *1.0*<br>*SC*[c]: *0.5*<br>*L*[d]: *0*
11	NPT	0.002 ps	100 ps	*BB*[b]: *0.5*<br>*SC*[c]: *0.1*<br>*L*[d]: *0*
12	NPT	0.002 ps	100 ps	*BB*[b]: *0.1*<br>*SC*[c]: *0*<br>*L*[d]: *0*
13	NPT	0.002 ps	10000 ps	*BB*[b]: *0*<br>*SC*[c]: *0*<br>*L*[d]: *0*

[a]Force constants are in kcal/(mol·Å^2)

[b]BB: Positional harmonic restraints on protein backbone and DNA heavy atoms

[c]SC: Positional harmonic restraints on protein side chains heavy atoms

[d]L: Harmonic restraints to keep the lipid tail in –5 Å<Z<5 Å, and lipid head groups close to the membrane surface (Z Z=±19 Å for POPC)

Supplementary Figures

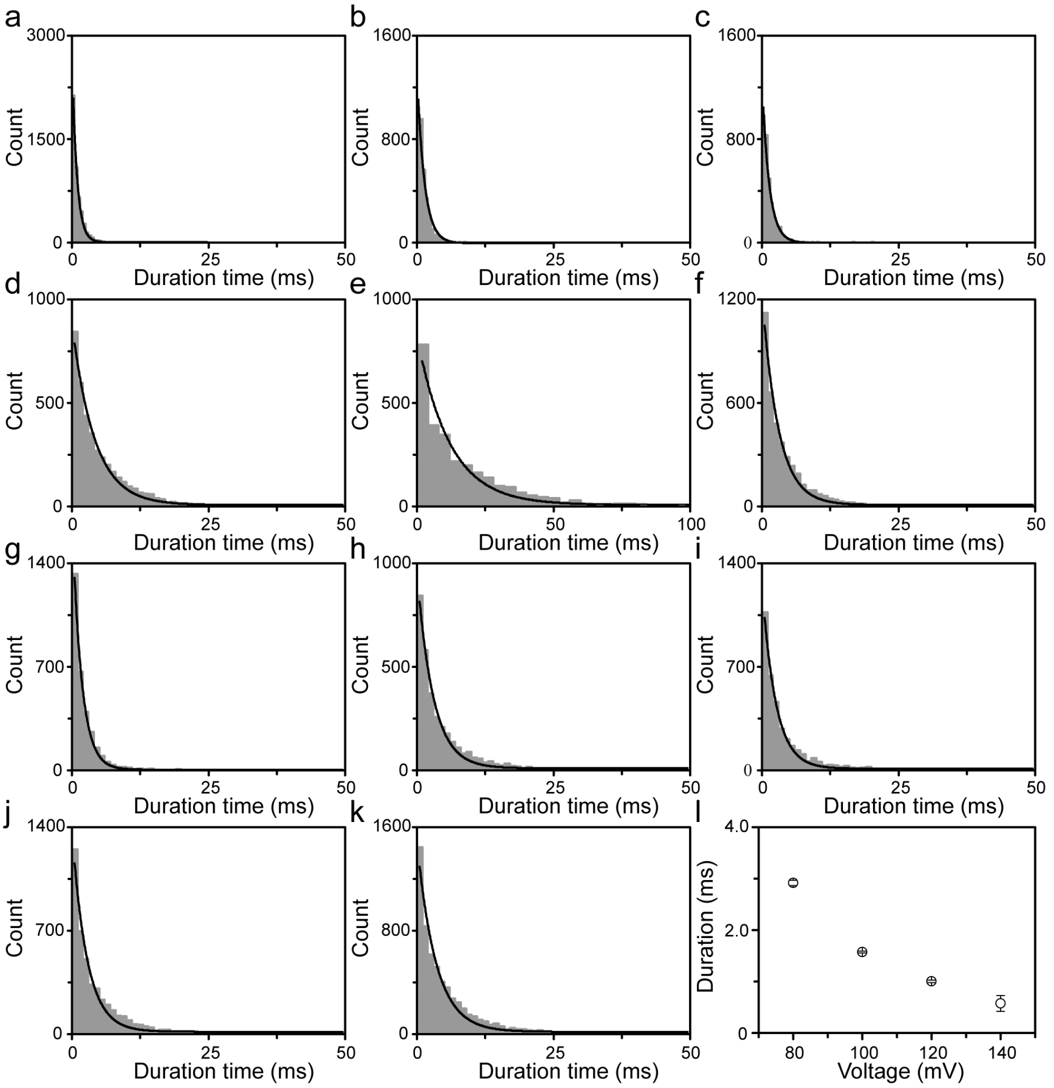

Supplementary Figure 1. Duration times of dA_n traversing through aerolysin nanopore. Histograms of duration times for **(a)** dA_{10}, **(b)** dA_{11}, **(c)** dA_{12}, **(d)** dA_{13}, **(e)** dA_{14}, **(f)** dA_{15}, **(g)** dA_{16}, **(h)** dA_{17}, **(i)** dA_{18}, **(j)** dA_{19} and **(k)** dA_{20} were all fitted to single-exponential distributions (solid lines) with the values of 0.58 ± 0.15 ms, 0.88 ± 0.15 ms, 0.85 ± 0.16 ms, 4.11 ± 0.56 ms, 11.91 ± 0.29 ms, 2.96 ± 0.78 ms, 1.70 ± 0.55 ms, 2.95 ± 0.23 ms, 2.48 ± 0.19 ms, 2.73 ± 0.48 ms and 3.45 ± 0.17 ms, respectively. **(l)** Effect of applied voltage on duration of dA_{10} with error bar based on at least 3 separated experiment. All data were obtained in 1.0 M KCl, 10 mM Tris, and 1.0 mM EDTA, pH=8.0, 24 ± 2 °C under the bias potential of + 140 mV. Each histogram contains more than 2000 events.

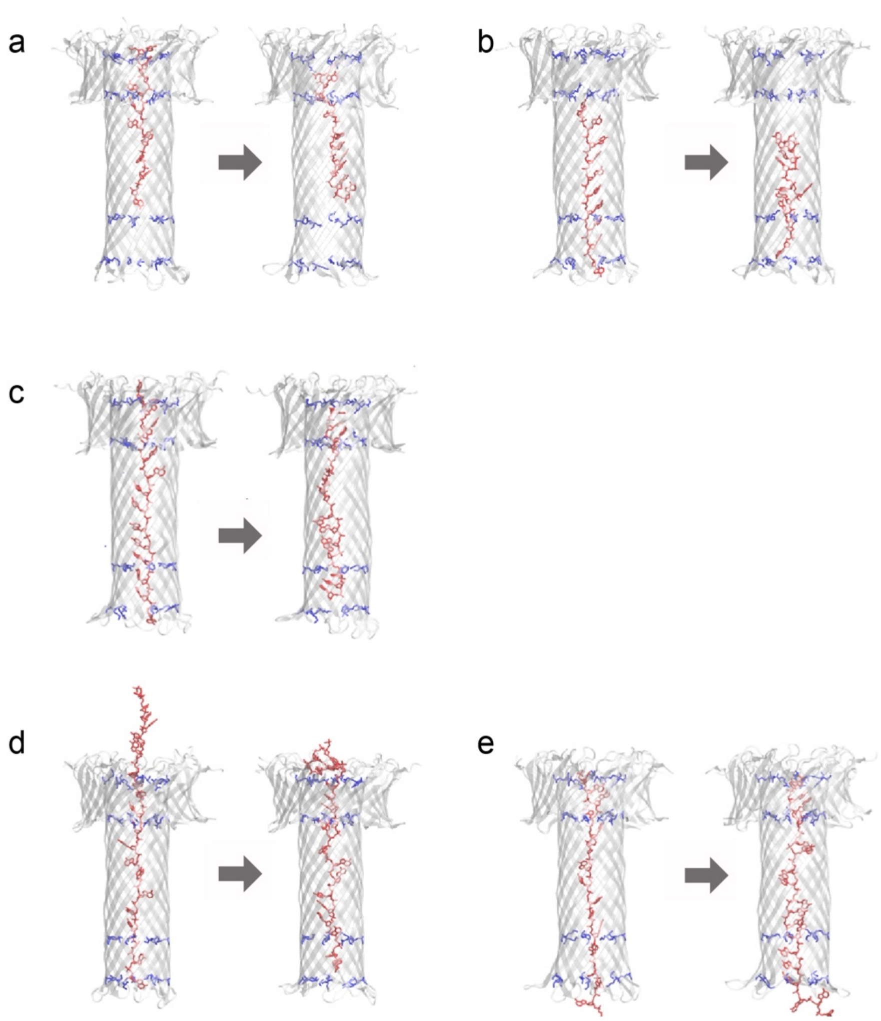

Supplementary Figure 2. Comparison of the fitting of dA_{10}, dA_{14} and dA_{20} in the pore. For each image, in the left it is represented the snapshot taken from the corresponding SMD, used as input for 150 ns of MD, with the average frame shown in the right. The positive residues R282, R220, K238 and K244 are shown as blue sticks, while the DNA is shown in red. (**a**) dA_{10} with the 5'-end at the pore entry, (**b**) dA_{10} with the 3'-end at the pore exit, (**c**) dA_{14} with the 5'-end at the pore entry, (**d**) dA_{20} with the 3'-end at the pore exit, (**e**) dA_{20} with the 5'-end at the pore entry and the 3'-end at the pore exit.

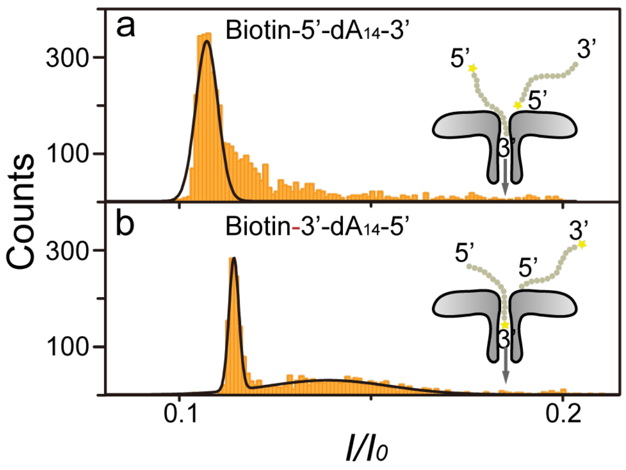

Supplementary Figure 3. Translocation of oligonucleotides with biotin modified at 5' or 3' end. Current blockage histograms with corresponding Gaussian fits (*Left*) and schematic diagrams (*Right*) for Biotin-5'-dA$_{14}$-3' (**a**) and Biotin-3'-dA$_{14}$-5' (**b**) traversing aerolysin pores. All data were acquired at 24 ± 2 ℃ with the applied voltage of 100 mV, in 3.0 M KCl, 10 mM Tris and 1.0 mM EDTA buffered at pH 8.0 in the presence of 5.0 μM oligonucleotide. Current was filtered at 5 kHz and sampled at 100 kHz.

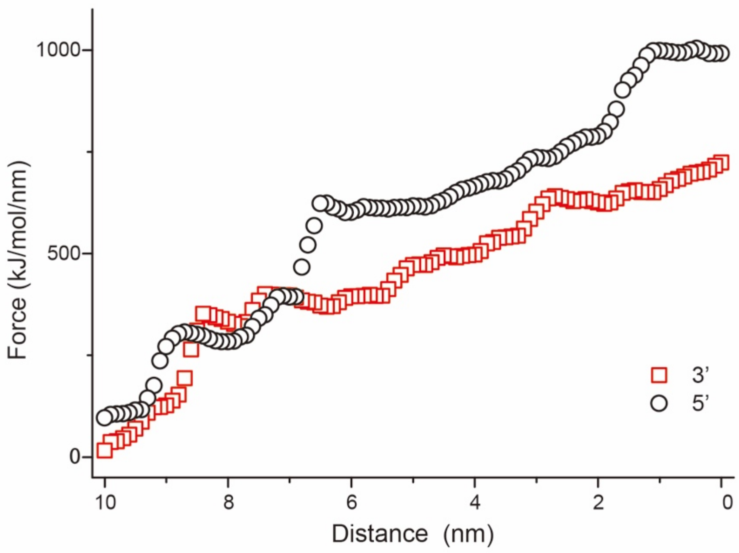

Supplementary Figure 4. SMD results of DNA across aerolysin pore. The force needed to decease the distance between the pushing group and the pore exit is shown, either applying a steering force by the 5' (black) or 3' (red) nucleotide.

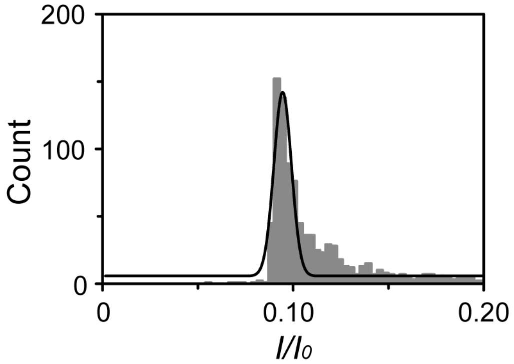

Supplementary Figure 5. Residual current level for $dA_{14}X1$. The translocation event histogram of residual current was fit by a Gaussian function (solid line). The data were obtained in 1.0 M KCl, 10 mM Tris, and 1.0 mM EDTA, pH=8.0, 24 ± 2 °C under the bias potential of + 140 mV.

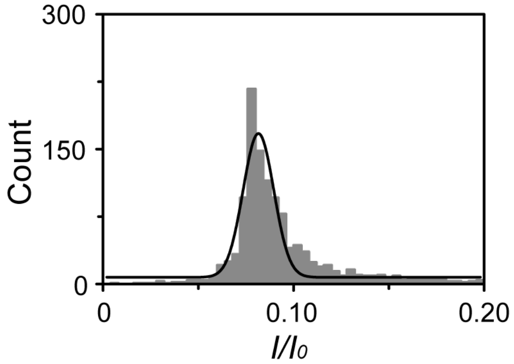

Supplementary Figure 6. Residual current level for $dA_{14}X2$. The translocation event histogram of residual current was fit by a Gaussian function (solid line). The data were obtained in 1.0 M KCl, 10 mM Tris, and 1.0 mM EDTA, pH=8.0, 24 ± 2 °C under the bias potential of + 140 mV.

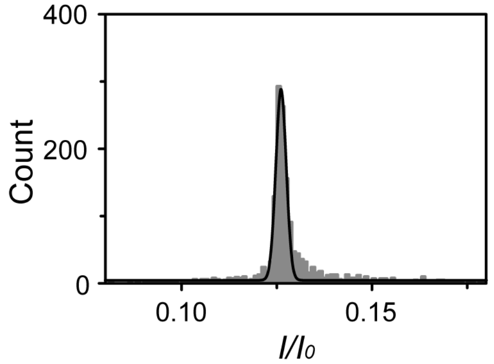

Supplementary Figure 7. Residual current level for $dA_{14}X3$. The translocation event histogram of residual current was fit by a Gaussian function (solid line). The data were obtained in 1.0 M KCl, 10 mM Tris, and 1.0 mM EDTA, pH=8.0, 24 ± 2 °C under the bias potential of + 140 mV.

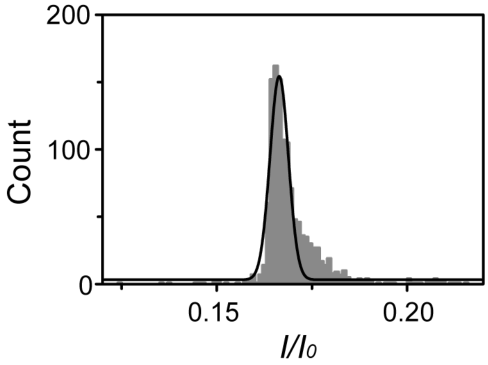

Supplementary Figure 8. Residual current level for $dA_{14}X4$. The translocation event histogram of residual current was fit by a Gaussian function (solid line). The data were obtained in 1.0 M KCl, 10 mM Tris, and 1.0 mM EDTA, pH=8.0, 24 ± 2 °C under the bias potential of + 140 mV.

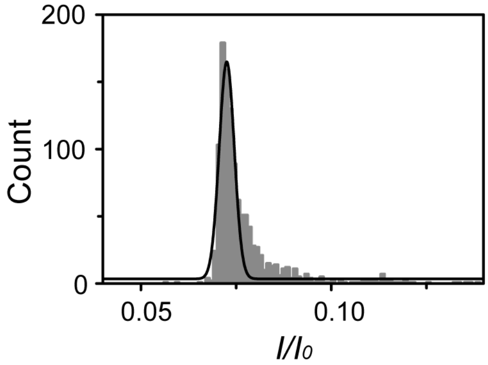

Supplementary Figure 9. Residual current level for $dA_{14}X5$. The translocation event histogram of residual current was fit by a Gaussian function (solid line). The data were obtained in 1.0 M KCl, 10 mM Tris, and 1.0 mM EDTA, pH=8.0, 24 ± 2 °C under the bias potential of + 140 mV.

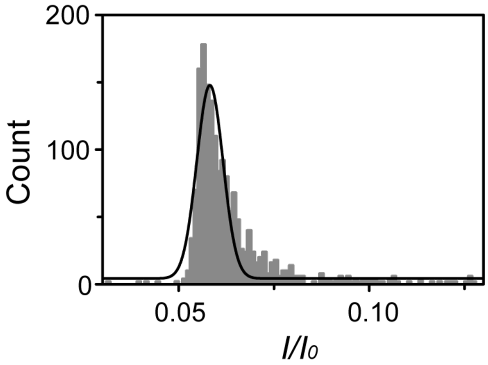

Supplementary Figure 10. Residual current level for $dA_{14}X6$. The translocation event histogram of residual current was fit by a Gaussian function (solid line). The data were obtained in 1.0 M KCl, 10 mM Tris, and 1.0 mM EDTA, pH=8.0, 24 ± 2 °C under the bias potential of + 140 mV.

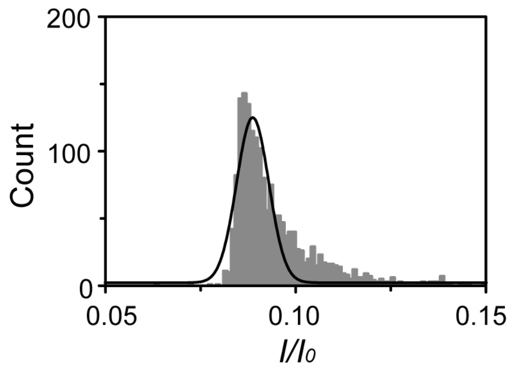

Supplementary Figure 11. Residual current level for dA$_{14}$X7. The translocation event histogram of residual current was fit by a Gaussian function (solid line). The data were obtained in 1.0 M KCl, 10 mM Tris, and 1.0 mM EDTA, pH=8.0, 24 ± 2 °C under the bias potential of + 140 mV.

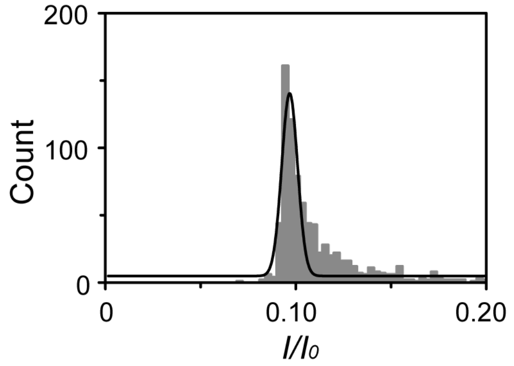

Supplementary Figure 12. Residual current level for dA$_{14}$X8. The translocation event histogram of residual current was fit by a Gaussian function (solid line). The data were obtained in 1.0 M KCl, 10 mM Tris, and 1.0 mM EDTA, pH=8.0, 24 ± 2 °C under the bias potential of + 140 mV.

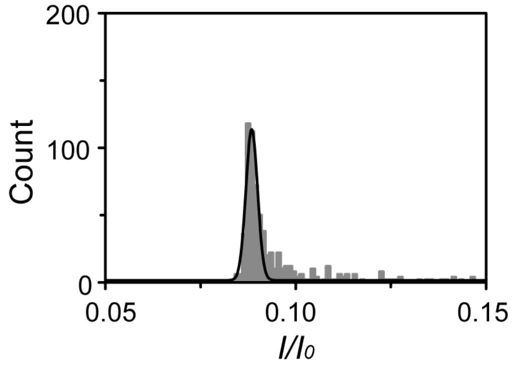

Supplementary Figure 13. Residual current level for dA$_{14}$X9. The translocation event histogram of residual current was fit by a Gaussian function (solid line). The data were obtained in 1.0 M KCl, 10 mM Tris, and 1.0 mM EDTA, pH=8.0, 24 ± 2 °C under the bias potential of + 140 mV.

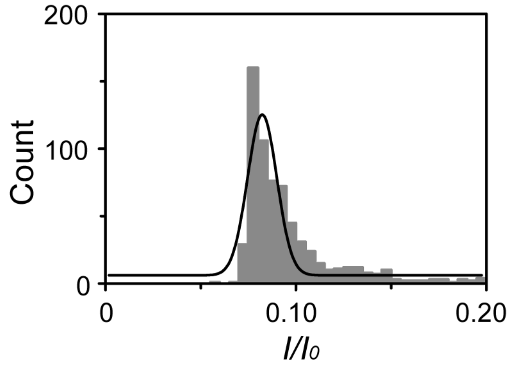

Supplementary Figure 14. Residual current level for $dA_{14}X10$. The translocation event histogram of residual current was fit by a Gaussian function (solid line). The data were obtained in 1.0 M KCl, 10 mM Tris, and 1.0 mM EDTA, pH=8.0, 24 ± 2 °C under the bias potential of + 140 mV.

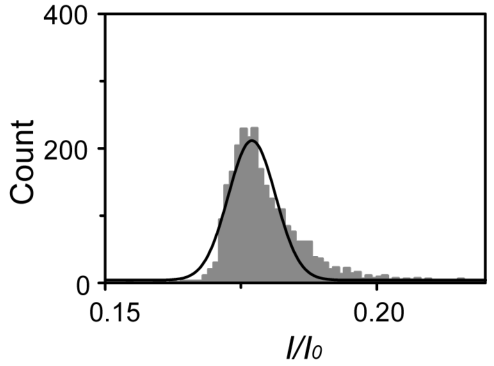

Supplementary Figure 15. Residual current level for $dA_{14}X11$. The translocation event histogram of residual current was fit by a Gaussian function (solid line). The data were obtained in 1.0 M KCl, 10 mM Tris, and 1.0 mM EDTA, pH=8.0, 24 ± 2 °C under the bias potential of + 140 mV.

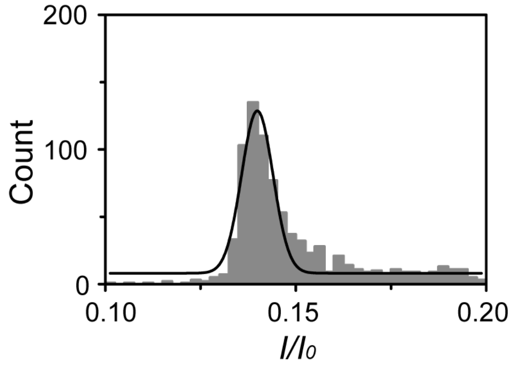

Supplementary Figure 16. Residual current level for $dA_{14}X12$. The translocation event histogram of residual current was fit by a Gaussian function (solid line). The data were obtained in 1.0 M KCl, 10 mM Tris, and 1.0 mM EDTA, pH=8.0, 24 ± 2 °C under the bias potential of + 140 mV.

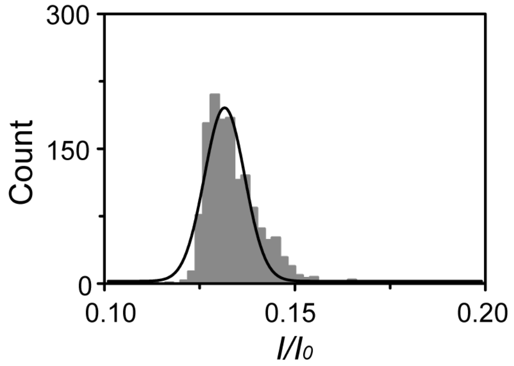

Supplementary Figure 17. Residual current level for $dA_{14}X13$. The translocation event histogram of residual current was fit by a Gaussian function (solid line). The data were obtained in 1.0 M KCl, 10 mM Tris, and 1.0 mM EDTA, pH=8.0, 24 ± 2 °C under the bias potential of + 140 mV.

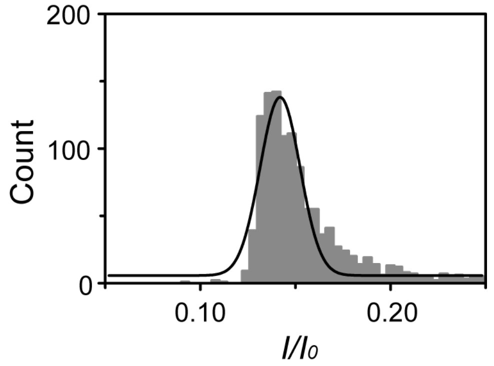

Supplementary Figure 18. Residual current level for $dA_{14}X14$. The translocation event histogram of residual current was fit by a Gaussian function (solid line). The data were obtained in 1.0 M KCl, 10 mM Tris, and 1.0 mM EDTA, pH=8.0, 24 ± 2 °C under the bias potential of + 140 mV.

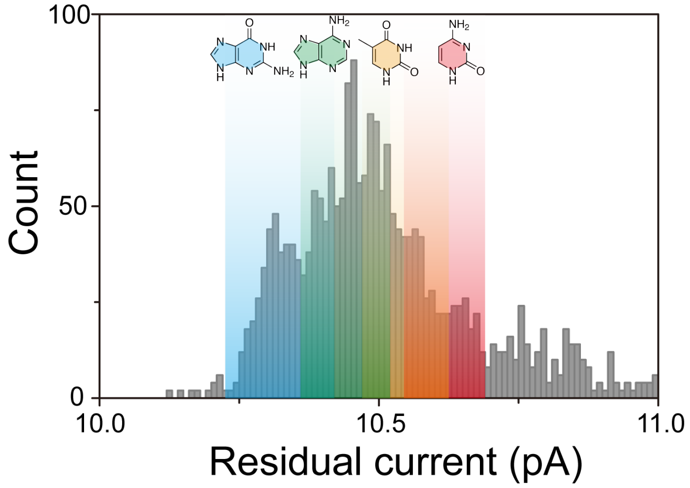

Supplementary Figure 19. Recognition of all 4 DNA bases by $dA_{14}X4$ sites of the aerolysin nanopore. WT aerolysin pore cannot discriminate single nucleobase differences at position 4(numbered from the 3'-end of oligonucleotides) according to histogram of residual current for the mixture of 4 poly $(dA)_{14}$ each containing either a single G, A, T, C nucleotide at position 4. The data were acquired in 3.0 M KCl, 10 mM Tris, and 1.0 mM EDTA, pH=8.0, 17 ± 2 °C under the bias potential of + 100 mV.

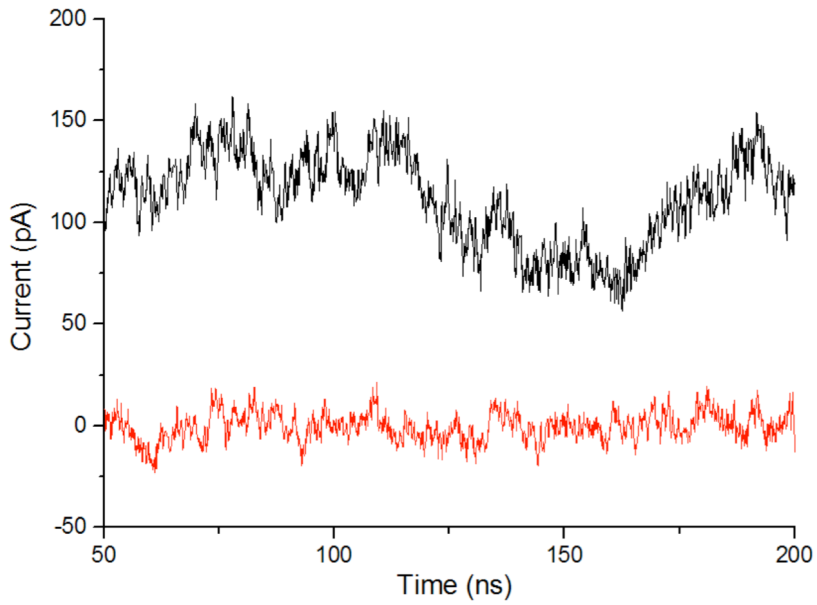

Supplementary Figure 20. Calculation of ionic current by MD simulations. Estimated current along the trajectory for a transmembrane aerolysin system at 1.0 M KCl under 250mV without (black) and with (red) dA_{14} translocating through the pore. The calculations were performed using established methods[3], and the average results of simulations are about 118.6 ± 1.4 and 0.7 ± 0.3 pA without/with DNA, respectively, which are consistent with the experimental values measured for the open pore (125.0 pA) and dA_{14}-bound aerolysin pore (5.0 pA). Absolute current values calculated by MD methods may be however overestimated by 10-40%.

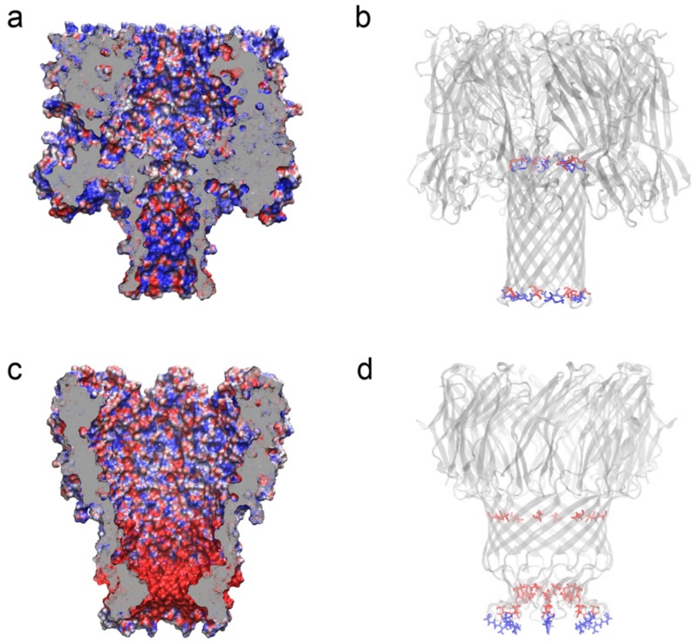

Supplementary Figure 21. Electrostatic surface of α-hemolysin and MspA nanopore. (**a**) Side view of the electrostatic potential of α-hemolysin. (**b**)The charged residues locating at the transmembrane of the α-hemolysin, from top to bottom, K147, E111, D127, and K131. (**c**) Side view of the electrostatic potential of MspA pore. (**d**) The charged residues locating at the transmembrane of MspA, from top to bottom, D118, D90, D91, D93 and R96. The calculation is based on PBEQ server and published PDB (α-hemolysin: 7AHL; MspA: 1UUN).

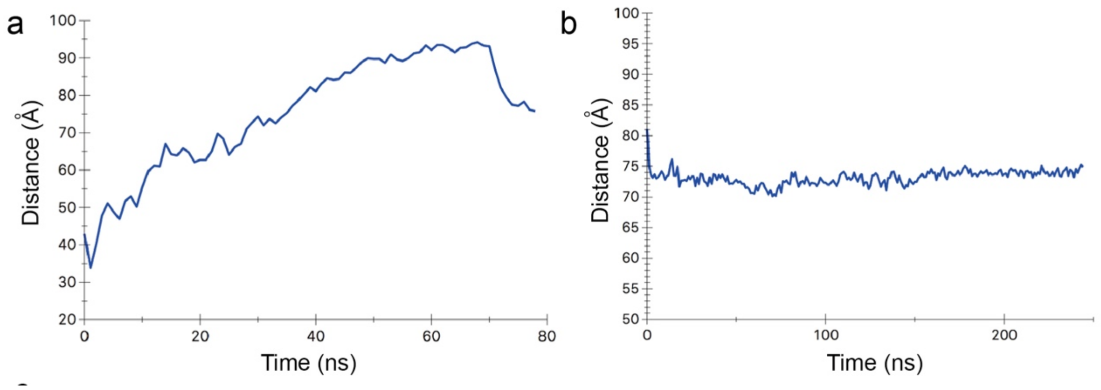

Supplementary Figure 22. Stretching DNA events. The ssDNA stretching has been measured as the distance between the first and the last phosphate atoms of dA_{14} along the following simulations: (**a**) During SMD, reaching a final distance of 9.5 nm when the whole DNA molecule is inside the pore. (**b**) During MD, where we observe a fast relaxation, reaching a distance of around 7.3 nm, which is lower than the expected contour length of dA_{14}, i.e., the entire length of a fully stretched ssDNA of 14 bases (8.3 – 9.8 nm).[1, 2]

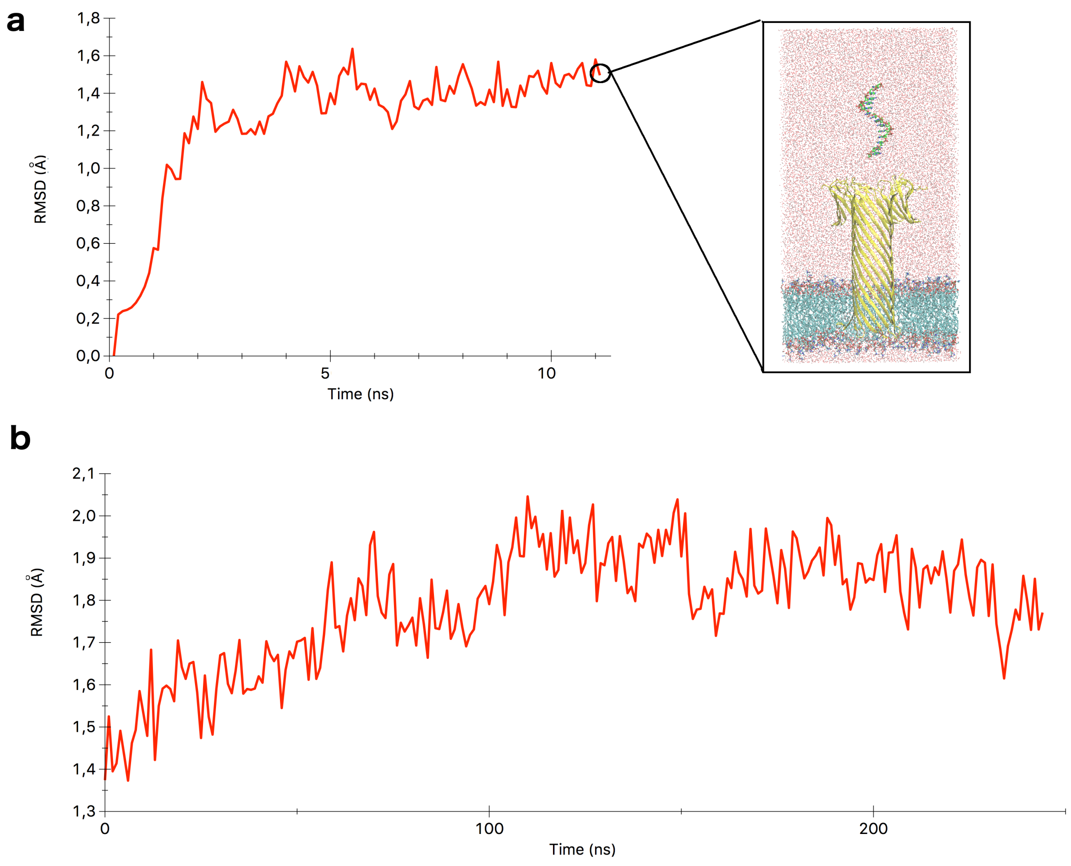

Supplementary Figure 23. Backbone Root Mean Square Deviation (RMSD). RMSD during the equilibration phase, including 10 ns unrestrained MD **(a),** and along the MD simulations with applied electric field **(b)**. As reference structure for the RMSD calculations, the original frame at the beginning of the equilibration was used. In the look-up, a representative snapshot showing the location of the protein and DNA with respect to the lipid bilayer, and the extent of the solvent volume at the end of the equilibration is reported. The protein is shown in yellow cartoon, the lipids as sticks with carbon atoms in cyan, and the DNA as sticks with carbon atoms in green. The box has been clipped from the front to improve visualization.

References

1. Olson, W. K. Configurational statistics of polynucleotide chains. A single virtual bond treatment. *Macromolecules*. **8**, 272–275 (1975).
2. Saenger, W. Principles of Nucleic Acid Structure. Springer-Verlag New York, (1984).
3. Aksimentiev, A.; Schulten, K. Imaging α-Hemolysin with Molecular Dynamics: Ionic Conductance, Osmotic Permeability, and the Electrostatic Potential Map. *Biophys. J.* **88**, 3745-3761 (2005).
